# Evaluation of U-Notch and V-Notch Geometries on the Mechanical Behavior of PVDF: The DIC Technique and FEA Approach

**DOI:** 10.3390/polym16202906

**Published:** 2024-10-15

**Authors:** Ingrid C. S. Pereira, José Renato M. de Sousa, Celio A. Costa

**Affiliations:** 1 Department of Metallurgical and Materials Engineering, COPPE/UFRJ, Federal University of Rio de Janeiro, Rio de Janeiro 21941-596, Brazil; ingrid_13@metalmat.ufrj.br; 2 Department of Civil Engineering, COPPE/UFRJ, Federal University of Rio de Janeiro, Rio de Janeiro 21941-596, Brazil; jrenato@laceo.coppe.ufrj.br

**Keywords:** polyvinylidene fluoride (PVDF), digital image correlation (DIC), finite element analysis (FEA), notch geometry

## Abstract

The notch effect of semicrystalline PVDF was investigated using U- and V-notch geometries with different depths, and tensile tests were performed at 23 °C using the DIC technique and FEA. Both unnotched and notched dumbbell-shaped specimens were subjected to tensile loading with the DIC technique to obtain mechanical curves and strain maps. The experimental data were compared to a numerical model, analyzing both global mechanical curves and local strain maps around the notch region to assess the accuracy of the simulations. The results demonstrated that the geometry and depth of the notch influence the mechanical behavior of PVDF, presenting a decrease in load and displacement compared to unnotched specimens. This aspect was corroborated by strain maps, which showed the increase in the local strain around the notch tip. For FEA, the global analysis indicated a good correlation with experimental results, and the local analysis demonstrated a reasonable agreement in strain map results within 0.5 mm of the notch neighborhood. Overall, the DIC technique and FEA provided a reliable evaluation of notch behavior on the PVDF used as pressure sheaths with reasonable precision.

## 1. Introduction

Notches are widely recognized as sources of stress concentration and have been extensively studied in various materials, focusing on factors like their shape (U- and V-notches), radius of curvature, and depth [1,2]. Hence, their presence is crucial in the design, fabrication, operation, and life assessment of several mechanical components, mainly due to their adverse impact on the material’s embrittlement factor, which favors crack nucleation and reductions in the component tensile strength and fatigue resistance [3].

Over the past few decades, studies have delved into the notch effect in polymers employing a variety of experimental methods, including holographic interferometry [4], photoelasticity [5], and digital image correlation (DIC) [6,7], as well as analytical/numerical solutions (i.e., fracture parameters [8], William’s equations [6,8], and finite element analyses (FEA)) [8]. However, these studies have utilized nonstandard notches under specific elastic or elastoplastic monotonic conditions [9,10,11]. Indeed, there is currently no consensus in the literature regarding whether specimen and notch geometry is sustainable for accurately assessing the notch effect in the mechanical behavior of polymers.

In recent years, applying the DIC technique and FE models has gained popularity in studying the mechanical behavior and the strain and stress fields related to the notch effect [12,13]. For example, Liu et al. [14] implemented the DIC method to measure deformation maps in notched hydrogels under tensile load, and the experimental results demonstrated a good agreement with an FE model. Similarly, Torabi et al. [15] and Bahrami et al. [16] assessed the fracture behavior of notched polymethyl–methacrylate (PMMA) using the DIC technique and FE analysis. Both studies reported a good correlation between results. 

Understanding the strains or stresses at the notch tip is essential in evaluating the mechanical behavior of notches in polymers applied in advanced industries, such as the offshore oil and gas industry [17,18]. Nonetheless, the current notch tests are limited to amorphous polymers that exhibit brittle and quasi-brittle fractures below glass transition temperature (*T_g_*), where the stress distribution field around the notch can be illustrated through equations that provide a linear elastic stress–strain relation [8,16,19]. As a result, a limited number of works use the DIC method to extract displacement/strain maps of the notch in semicrystalline polymers. In particular, semicrystalline polyvinylidene fluoride (PVDF) layers are often employed as pressure sheaths for offshore flexible pipes [20]. These pipes comprise concentric metallic and polymeric layers. The metallic layers resist the imposed mechanical loads, while the polymeric layers ensure fluid tightness and/or reduce friction and wear between the metallic layers. Regarding the polymeric layers, the pressure sheath internally seals the pipe, ensuring that the transported fluid leaks to the outer water environment [18].

A study conducted by Hund et al. [21] investigated PVDF using CNBR geometry and the DIC method to predict voids during tensile loading. It was observed that yielding was influenced by the stress triaxiality effect caused by a dual notch of CNBR geometry. Moreover, Ychisawa [22] applied the DIC technique to evaluate blunt notches made in the PVDF (S_pb_ method) but encountered difficulties creating displacement maps due to the complexity generated by significant deformation neighborhood defects. Pereira [9] studied the effect of two types of notches and cracks using strain maps during tensile tests from commercial grades of PVDF to analyze large deformations and utilized finite element analysis (FEA) for modeling purposes.

The present research aims to investigate the impact of U-notch and V-notch geometries on stress concentration in a commercial-grade PVDF using DIC and FEA. For the first time, dumbbell-shaped PVDF specimens with different notch depths were subjected to tensile tests at room temperature, and strain maps around the notch tip and tensile curves were obtained. Finally, the experimental results from the tensile tests were compared with those from numerical simulations to evaluate the mechanical behavior of notched PVDF specimens.

## 2. Materials and Methods

### 2.1. Experimental Methodology

#### 2.1.1. Material and Specimen Preparation

**Figure 1:**
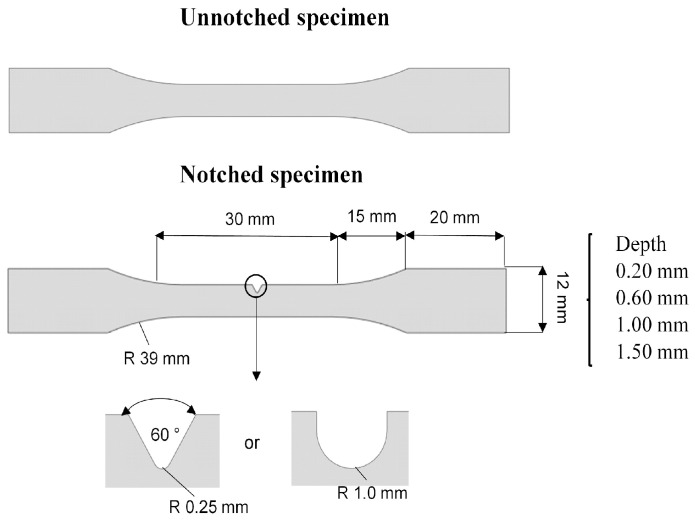
Illustration of unnotched and notched specimens.

The material studied was the PVDF with 3% plasticizer. Dumbbell-shaped (DBS) specimens with a constant thickness of 6 mm were machined in the longitudinal direction from commercially extruded pipes based on ISO 527-2 (Type 5A) [23]. Unnotched samples were compared to U-notch samples (curvature radii of 1 mm) and V-notch samples (curvature radii of 0.25 mm, and internal angle of 60°) with varying notch depths (0.2 mm, 0.6 mm, 1.0 mm, and 1.5 mm) to evaluate the notch effect. Figure 1 and Table 1 illustrate the specimens’ notch geometries and test matrix, respectively. 

The specimens were coated with black spray paint to create a random speckle pattern, allowing DIC measurements during the tensile tests. This coating has a unique speckle pattern that contrasts the black dots and the natural white surface of PVDF, facilitating the digital sensor’s recognition of surface displacement during load application. The black covered area fraction *Fr* (%) and the speckle size through Ferret diameter *D_F_* were measured using ImageJ [24] software (version 1.54d) to evaluate the quality of the speckle pattern. These values were also compared to the image processing of painted samples presented in a previous study [9].

#### 2.1.2. Experimental Setup of DIC

Five samples were tested per specimen condition to verify the reproducibility of the results obtained by mechanical tests. Uniaxial tensile tests combined with DIC were conducted until specimen fracture at room temperature (23 °C) under 5 mm/min displacement control, employing an electromechanical universal testing machine (INSTRON 5567) with a 10 kN load cell. Thus, experimental data were reaction forces measured by the load cell, displacements obtained from tensile control, and the strain maps plotted using the DIC system. For comparison, tensile tests were also conducted using a 25 mm extensometer to assess the accuracy of the DIC technique.

**Figure 2:**
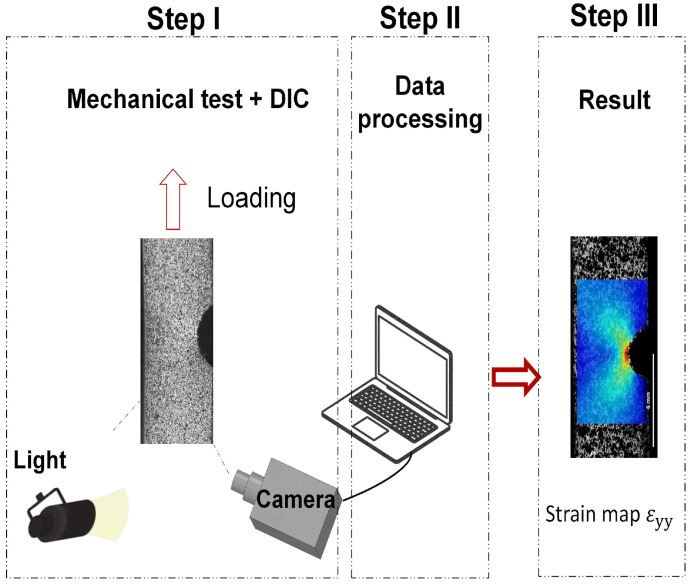
Schematic of 2D-DIC setup: Step I—acquisition of images, Step II—post-processing, and Step III—the resulting strain map.

Throughout the experiment, optical images of the specimens’ side surfaces were captured using a digital camera equipped with a 5MP resolution and a 100 mm Tokina lens. A light device and a computer were utilized to ensure image registration. The eCorr lab [25] acquisition software facilitated image acquisition at a speed of five frames per second (5Hz), with each DIC acquisition frame correlated with the load and displacement obtained by Blue Hill 3. The camera was positioned at 1.30 m for the unnotched specimens and 0.27 m for the notched samples. The Ncorr v.1.2 software [26], which runs on MATLAB R 2020a, was used to post-process the images. Figure 2 displays the experimental setup employed for the acquisition of images (Step I) and post-processing (Step II) to obtain strain maps (Step III).

#### 2.1.3. FE Model Implementation

In this study, a finite element (FE) model was employed to simulate the behavior of notches in PVDF samples subjected to monotonic tensile tests. The FE model was two-dimensional (2D), representing the geometry of the tested samples, and was developed in ANSYS^®^ [27]. As several FE meshes were employed in this study, their construction was automatized in a macro written in APDL (ANSYS Parametric Design Language), allowing their pre-processing, solution, and post-processing. 

**Figure 3:**
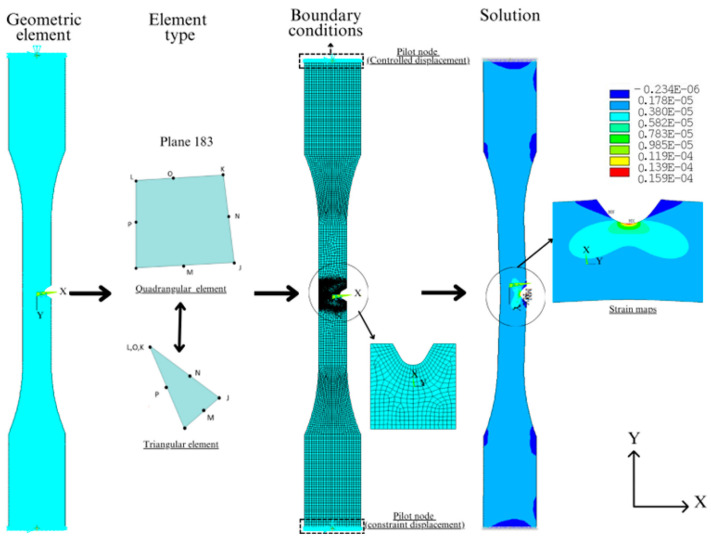
Element finite model showing the tensile test, mesh generation, and boundary conditions in the numerical simulations.

The notched tensile specimens of different depths were modeled with 8-node quadrangular plane stress finite elements, named PLANE183 in ANSYS^®^. Each node has two degrees of freedom, i.e., translations regarding the X and Y directions. Moreover, a triangular degenerated form with six nodes was employed in regions with irregular geometry. A view of a typical FE mesh is presented in Figure 3. PLANE183 has quadratic interpolation functions suitable for modeling regions with high-stress gradients, such as those found near notches, justifying its choice instead of elements with linear interpolation functions. For instance, Thirumump et al. [28] employed this element to evaluate stress concentrations in plates with holes, obtaining satisfactory results.

**Figure 4:**
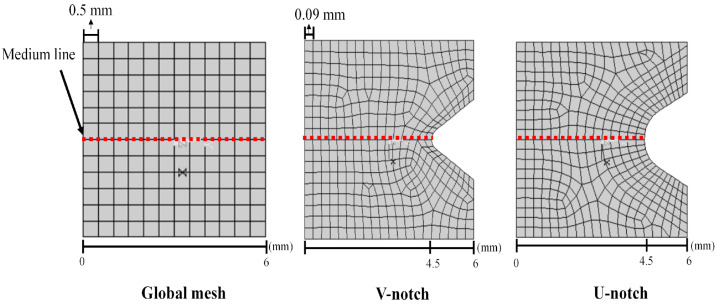
Illustration of a close-up to observe global mesh and mesh in the notch region.

Figure 4 shows a close-up view of the mesh configuration used in the FE model. As a result of the high stress at the notches’ tips, an unstructured refined mesh was adopted in the vicinity of the notch neighborhood (V-notch or U-notch), whereas a coarser structured FE mesh was constructed following the specimen geometry in regions far from the notches. Moreover, the red dashed line corresponds to the medium line on which the strain values were extracted for later comparison with DIC results.

Additionally, the constructed FE models aimed to simulate the failure of the PVDF samples. Hence, large strains and material nonlinearities were expected. Geometric nonlinearities, such as large strains, are directly accounted for in PLANE183. In contrast, this work assumed a rate-independent material response, i.e., viscoelastic effects were disregarded. Hence, the material constitutive model required defining a stress vs. strain relation by considering a yield (failure) criterion, a hardening rule, and a flow rule. The relation between the uniaxial stress state and the multiaxial stress state induced in the tests was established with the von Mises yield criterion, assuming PVDF as a ductile material. Moreover, the imposed load was monotonic, and isotropic hardening was considered, following the recommendation presented in ANSYS^®^ (version 19.2) [27]. Finally, the flow rule was associative, i.e., the potential was identical to the von Mises yield surface.

**Figure 5:**
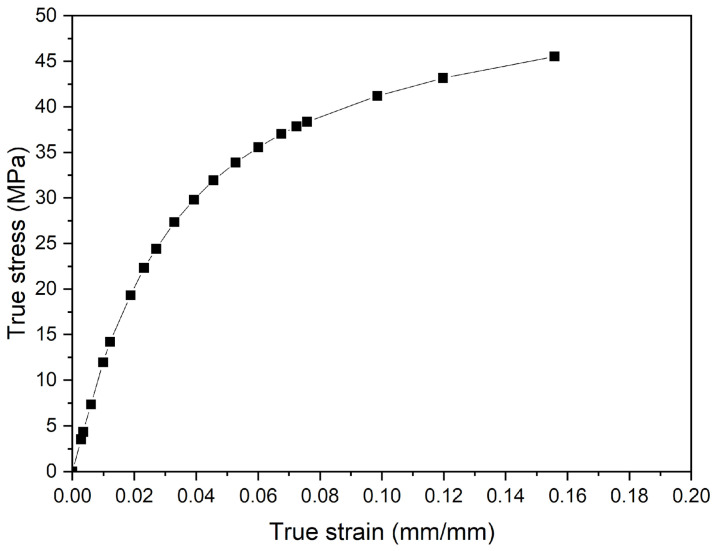
True stress–strain curve of PVDF adjusted to multilinear model.

Table 2 and Figure 5 present the input of material properties in the numerical model. The true stress–strain curve’s components were calculated using Equations (1) and (2) [29], which were adjusted to the multilinear model from the stress–strain curve of the unnotched samples obtained by tensile tests with the DIC technique.

(1) $$\sigma_{true}=\sigma_{eng}(\sqrt{2\times \varepsilon_{yy-lag}+1})$$

(2) $$\varepsilon_{true}=In(\sqrt{2\times \varepsilon_{yy-lagg}+1})$$

where ε_yy_ is the Lagrangian strain in the longitudinal direction (Y direction).

To simulate the tensile test, the geometry and boundary conditions identical to those imposed on the experimental tensile tests were selected. The boundary conditions were applied at pilot nodes positioned at the ends of each model. The pilot nodes were meshed with CONTA175 elements and were rigidly connected to the nodes on the edge lines of the sample, which were meshed with TARGE170 elements. Hence, rigid contact pairs were formed, and a translation in the Y-axis direction was applied at the pilot node at one end of the specimen. The other end was constrained in the X and Y directions to avoid rigid body motion.

Furthermore, Pereira [9] conducted an FE mesh study to ensure the FE model’s accuracy, indicating that a maximum element edge length of 0.5 mm was required for the structured region. In comparison, a maximum length of 0.09 mm was demanded for the notch region. Table 3 presents the number of elements and nodes for each type of notch geometry.

Lastly, all numerical analyses were carried out using the sparse solver of ANSYS^®^ [27], employing automated load steps. At each load step, the convergence was achieved if the L2 norms of the residual forces, and moments were less than 0.1% of the absolute value of the acting total forces and moments. The first load step corresponded to 0.1% of the total imposed load, but depending on the convergence rate, this load step may be increased to 10% of the total imposed load or reduced to 0.01%. Considering all these aspects, a typical FE analysis was concluded after 2.1 h in an Intel i7 CPU and 16 GB of RAM.

## 3. Results and Discussion

### 3.1. Tensile Behavior of Unnotched Specimens: DIC and Extensometer

**Figure 6:**
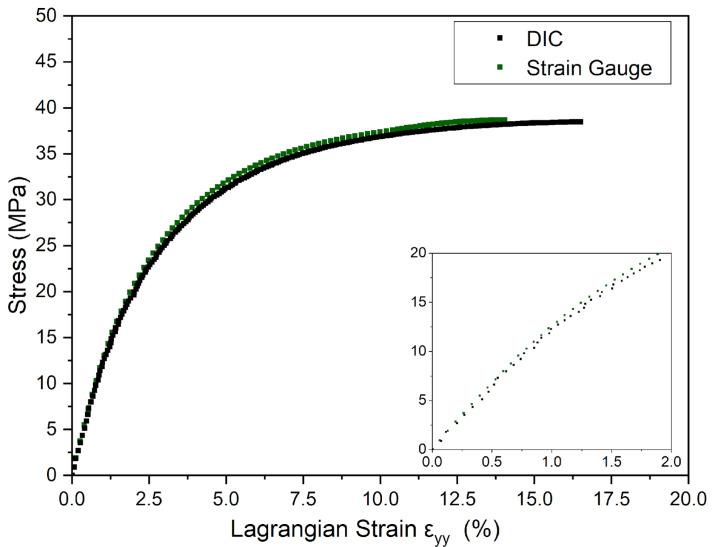
Comparison between stress versus Lagrangian strain εyy curves of DIC and strain gauge at 23 °C under the rate of 5 mm/min of unnotched specimens.

Mechanical tests of the unnotched specimens of PVDF were performed to obtain material properties for the FE model and investigate the accuracy and reliability of the DIC technique, as described in Section 2.1.2. The tests’ results revealed that the stress–strain curves generated with both methods were almost identical until the yield point, as seen in Figure 6, indicating a good agreement considering the expected viscoelastic behavior of semicrystalline polymers [30]. The yield strain measured from stress–strain curves using the extensometer was 14.5 ± 0.21%, while with the DIC method, it measured 16.2 ± 0.29%. This slight discrepancy can be attributed to two factors. Firstly, the extensometer, due to being a contact technique, may have introduced an unwanted stress concentration at the surface of the specimen, thus predicting the necking nucleation. Secondly, despite its efficiency in obtaining reliable and accurate strain data, the accuracy of the DIC in measuring strain values depends on several factors, such as the speckle pattern quality, the experimental setup, and the degree of noise during loading, which may affect the accuracy of the partial derivatives functions applied to calculate the strains, according to Blabber, Adair, and Antoniou [26].

**Figure 7:**
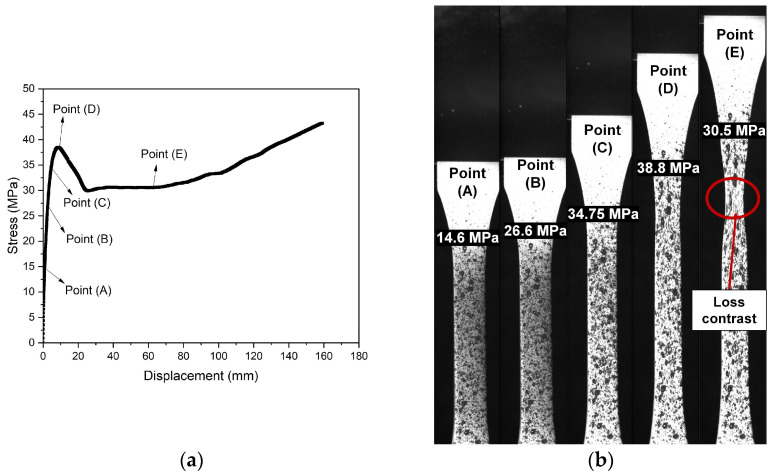
Illustrations (**a**) stress versus time with (**b**) images during tensile test using DIC to specific points: (A) stress at the linear–elastic region, (B) stress at 3.5% of strain, (C) stress at 7.0% of strain, (D) stress at the yield point, and (E) stress at necking.

The material’s mechanical response was analyzed through tensile tests using DIC’s images without post-processing. In Figure 7, the load values associated with local maximum strain at the notch tip are presented as follows: Point (A) at 1.2%, Point (B) at 3.5% (related to flexible pipe’s dynamic operation [31]), Point (C) at 7.0% (related to flexible pipe’s static operation [31]), and Point (D) at 16.2% (corresponding to the yield point criterium). Figure 7a shows that the material exhibited an apparent linear elastic behavior until the yield point (Point (D)), followed by a decrease in stress from around 38.9 MPa to 30.5 MPa at Point (E) once necking occurred. The stress remained constant (30.5 MPa) during the neck propagation. The PVDF sample demonstrated a ductile behavior associated with yielding and neck nucleation with the whitening phenomenon, which has also been reported by Defebvin et al. [32] and Castagnet et al. [33,34]. Figure 7b shows this phenomenon in the neck region, indicating a cavitation process followed by void coalescence in the samples. The whitening presented at unnotched samples led to the contrast loss of speckle pattern in the surface of the specimens under tensile test using the DIC at high strains. To ensure reliable and accurate data, the strain maps extracted until the yield point (dσ/dε = 0, necking locus [35]) were compared, as illustrated until Point (D). 

### 3.2. Strain Map Distribution for Unnotched Specimens

**Figure 8:**
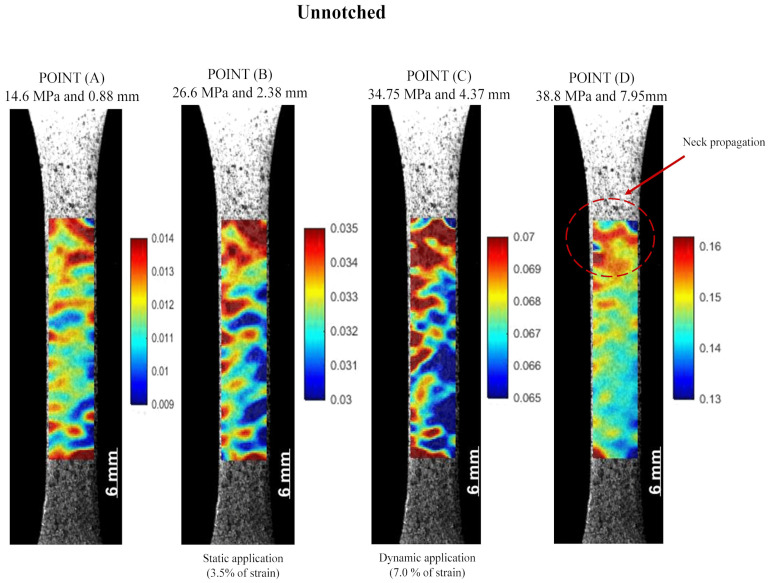
Lagrangian strain εyy maps of unnotched specimens that correspond to specific points of Stress versus Lagrangian strain εyy curve: (A) stress at the linear–elastic region, (B) stress at 3.5% of strain, (C) stress at 7.0% of strain, and (D) stress at the yield point.

One of the significant advantages of the DIC is the ability to generate strain maps on the specimen surface. Figure 8 shows the typical strain maps obtained from the DIC for specific stresses in the stress–time curve shown in Figure 7a. At Point (A), an increase in the deformation is observed, with a homogenous Lagrangian strain in the Y-axis ($\varepsilon_{yy}^{Lag}$) distribution in the region of interest (ROI), i.e., below 1.2%. The values observed in the strain maps when PVDF reached 3.5% are noteworthy (27.2 ± 0.4 MPa, Point (B)) and 7.0% (34.6 ± 0.3 MPa, Point (C)) strains, which are associated with the limit strains for PVDF operation under dynamic and static operation as a pressure sheath according to API SP 17J [31], respectively. Point (D) corresponds to the yield point, which, on average, corresponds to a stress of 38.9 ± 0.5 MPa and a strain of 16.2 ± 0.29%, where the red mark shows the area that nucleated the neck. The DIC accurately measured the developed large strains. 

**Figure 9:**
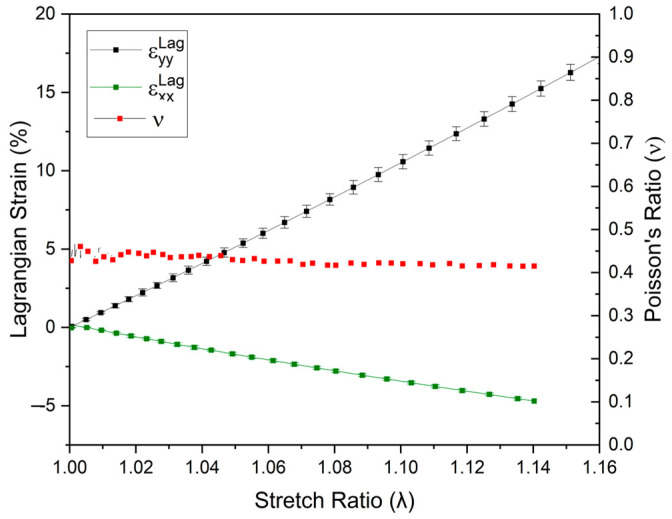
Poisson’s ratio (v) and longitudinal ($\varepsilon_{yy}^{Lag}$) and transversal ($\varepsilon_{xx}^{Lag}$) strains obtained using the DIC at different stretch levels until the yield point for the unnotched PVDF specimen.

The DIC technique also enabled measuring the deformation in both longitudinal and transversal directions until the yield point, as shown in Figure 9, indicating a negative $\varepsilon_{xx}^{Lag}$ due to the reduction in the transversal section and an increase in the longitudinal section ($\varepsilon_{yy}^{Lag}$ > 0), as expected in tensile tests. Furthermore, as the components $\varepsilon_{yy}^{Lag}$ and $\varepsilon_{xx}^{Lag}$ were also obtained in the elastic region, the Poisson coefficient *v* could also be determined. At a strain rate of 5 mm/min and a temperature of 23 °C, the mean value of *v* was found to be 0.43 ± 0.04. It is important to note that this average value is only valid for the specific test conditions because *v* is a time-dependent property for polymers, opposing what is observed in metallic materials [36]. Compared to the literature, the values of *v* obtained for PVDF (0.43 ± 0.04) were similar to those reported by Castagnet et al. [34], i.e., 0.36 to 0.47.

### 3.3. Tensile Behavior of Notched Specimens

**Figure 10:**
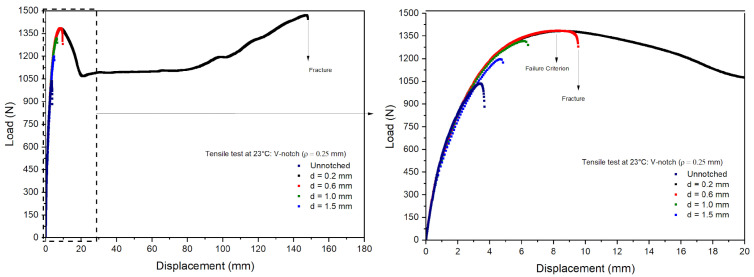
Load–displacement curves for V-notch specimens.

**Figure 11:**
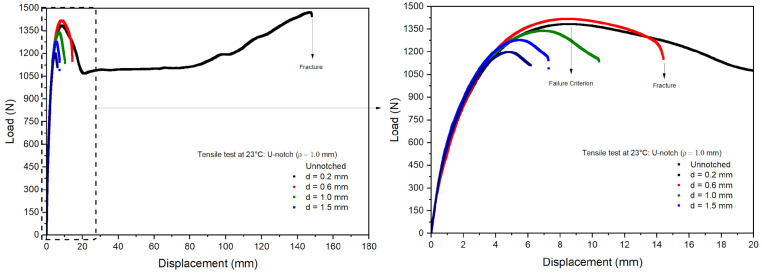
Load–displacement curves for U-notch specimens.

Tensile tests were performed on the notched specimens of PVDF under the same conditions as the unnotched tests. Figure 10 and Figure 11 show the measured load vs. displacement curves, evidencing nonlinear responses in all the notched specimens. The curves obtained for the notched specimens were close to the unnotched ones up to the associated failure loads. However, the failure loads of the notched specimens were lower than those observed in the unnotched specimens, and a significant decrease was also observed with an increase in notch depths. The failure of the notched specimens is related to stress concentrations around the notch root, causing local plastic deformations and the rupture of the specimens. As a result, the PVDF ductility perceived in the unnotched tests was significantly reduced, as observed, e.g., in polyolefins [37].

Table 4 presents the average maximum loads and displacements measured in the notched specimens’ tests normalized by the unnotched values. First, the results indicated good repeatability with small standard deviations, thus leading to coefficients of variation lower than 5.0%. For a notch depth of 0.2 mm, the V-notch samples exhibited values similar to the unnotched samples, while the U-notch samples with a depth of 0.2 mm had slightly higher values than the unnotched samples, possibly due to a local hardening effect. The loads and displacements decreased linearly for notch depths higher than 0.6 mm. The U-notch load drop was less steep than observed in the V-notch specimens, as the stress concentration increased at the notch root with decreasing curvature radii [38]. 

Furthermore, the impact of notches on the tensile properties can be understood by examining the PVDF micro-mechanisms. Shear flow or yielding and cavitation processes, which compete during the tensile tests, influence the fracture of PVDF above *Tg* [33,34]. The yielding process occurs uniformly in unnotched samples and is simultaneous to the whitening onset. On the other hand, the notches significantly alter the mechanical response of the samples due to the modified local stress state. Significant hydrostatic stress is ahead of the notch when cavitation tension reaches a critical value. As a result, notching constrains plastic yielding, and elastic performance and cavitation processes dominate at the notch root. Moreover, stress whitening around the notches is caused by these cavities, similar to what was observed in other PVDF specimens [39,40]. 

### 3.4. Strain Maps for Notched Specimens Using the DIC Technique

The DIC analysis was conducted in the notched specimens to assess the nucleation of the plastic zone at the notch tip, where damage was presumed to be significant. For comparison purposes in this study, the reference points are the same used in Figure 8. Then, the maximum strain measured was carried out up to the strain near the notch region to reach the yield strain to the unnotched specimens (Point (D) ~16%).

**Figure 12:**
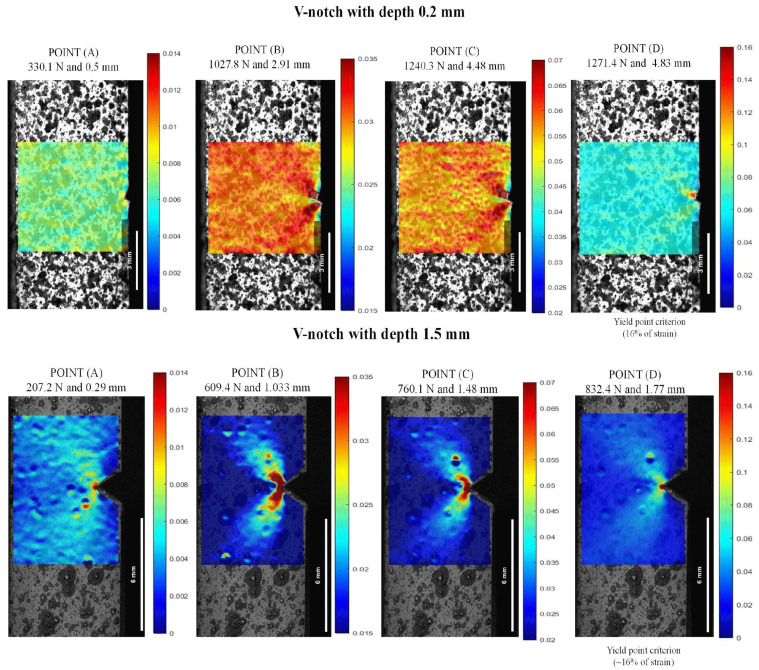
Lagrangian strains ε_yy_ in V-notch tests at 23 °C and 0.2 and 1.5 mm depths: (A) stress at the linear–elastic region, (B) stress at 3.5% of strain, (C) stress at 7.0% of strain, and (D) stress at the yield point.

**Figure 13:**
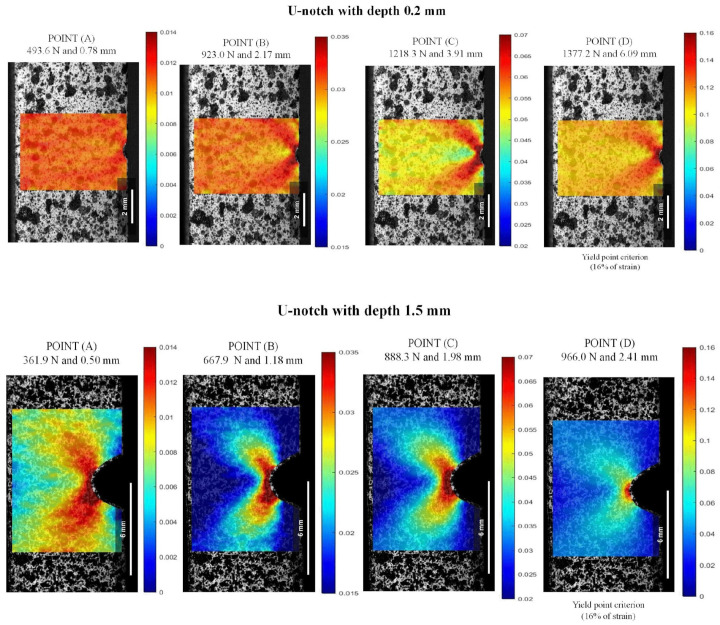
Lagrangian strains ε_yy_ in U-notch tests at 23 °C and 0.2 and 1.5 mm depths: (A) stress at the linear–elastic region, (B) stress at 3.5% of strain, (C) stress at 7.0% of strain, and (D) stress at the yield point.

Figure 12 and Figure 13 present the strain maps for depths of 0.2 mm and 1.5 mm in V- and U-notch geometries, respectively, as examples. There is a clear tendency toward strain accumulation in the notch region with the surrounding material at lower deformation, as demonstrated when comparing Point (A) to Point (B), where localized deformation increased by around 2.3%. Additionally, considering API 17J [31], the limit strain in the pressure sheath of a flexible pipe (dynamic operation) is 3.5%, and for a flexible flowline (static operation), it is 7.0%. These values correspond to the maximum deformations in the notch front at Point (B) and Point (C), respectively, and were observed before reaching the maximum load in all the notched conditions. 

As illustrated in the strain maps for the V-notch sample (Figure 12), increasing the notch from 0.2 to 1.5 mm led to a significant intensification of local stress concentration compared to the unnotched specimens. At a depth of 0.2 mm, the specimen presented a discrete local stress concentration, indicating displacements of 2.91 mm at 1027.8 N for Point (B) and 2.83 mm at 1240.3 N for Point (C). On the other hand, as the depth increased to 1.5 mm, the strain at the notch tip showed a local strain more spread out from the notch with a butterfly wing format (BWF), resulting in a reduction of around 35.0% for displacement and 59.5% for load, concerning 3.5% (Point (B)) and 7.0% (Point (C)). The increase in local strain concentration occurs because the elastic bulk material severely constrains the plastic deformation zone ahead of the notch for all strain fields. As a result, the area far from the defect zone experiences lower and more uniform deformation values [1,9]. This pattern was also observed in the U-notch specimens in Figure 13, where there was around a 49.3% decrease in displacement and approximately 27.6% decrease in load at Point (B) and Point (C) from 0.2 to 1.5 mm depth. 

As depicted in the strain maps for the U-notch (Figure 13), different notch geometries induced a distinct local strain concentration profile, thereby altering the mechanical behavior of PVDF, as seen in Figure 10 and Figure 11. U-notch specimens presented a damage zone spread far into the bulk compared with V-notch specimens, and V-notch tip blunting was limited. Indeed, for notches with the same depth, a smaller curvature radius at the notch root resulted in a lower load needed to achieve the same strain. For instance, at a depth of 0.2 mm and 1.2% strain, a V-notch required a load of 330.1 N, while a U-notch required 493.6 N. In this case, deformation was evenly distributed in both areas. As the load increased from Point (B) to Point (D), the V-notch concentrated all the strain at its tiny tip, while the U-notch was able to spread the deformation zone in a BWF. As shown in Figure 12, for a 1.5 mm depth, the V-notch concentrated the highest strain at its tip up to Point (C), and above it, the highest deformation zone spread from the tip in a BWF. On the other hand, as shown in Figure 13, the U-notch with 1.5 mm depth spread the BWF deformation zone up to Point (C) and then concentrated it at its tip, showing an opposite behavior.

### 3.5. FEA of Notched Specimens

The modeling results of the PVDF notches’ behavior using the finite element analysis (FEA) were compared to the experimental results to verify the proposed numerical model’s suitability and accuracy. 

**Figure 14:**
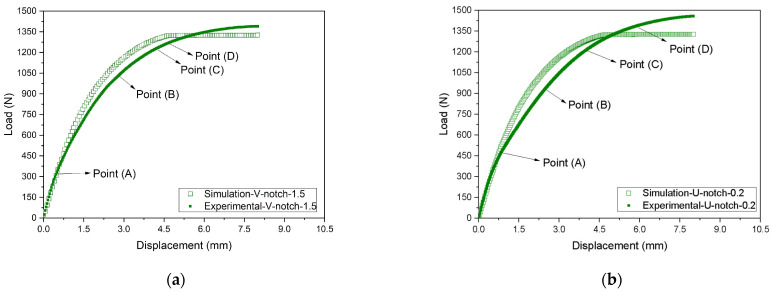
Comparison between experimental and FE responses considering notches with 0.2 mm depth in (**a**) V-notch and (**b**) U-notch samples: (A) stress at the linear–elastic region, (B) stress at 3.5% of strain, (C) stress at 7.0% of strain, and (D) stress at the yield point.

**Figure 15:**
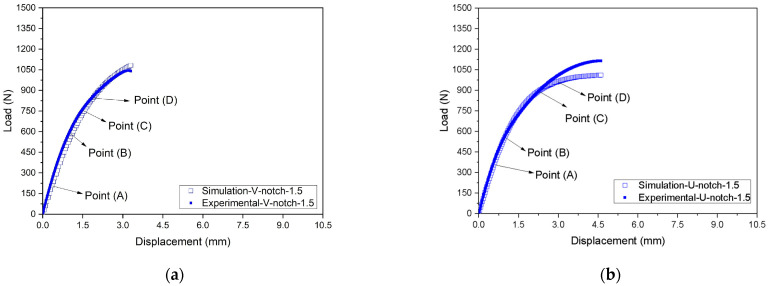
Comparison between experimental and FE responses considering notches with 1.5 mm depth in (**a**) V-notch and (**b**) U-notch samples: (A) stress at the linear–elastic region, (B) stress at 3.5% of strain, (C) stress at 7.0% of strain, and (D) stress at the yield point.

Figure 14 and Figure 15 and Table 5 compare the experimental and simulated load–displacement curves for both types of notches at all depths and considering the points indicated in Figure 8. The numerical model well captured the global behavior of the notched specimens. The numerical curves followed the experimental ones until reaching a plateau, indicating the samples’ rupture. As the notch depth increased, the displacement associated with the beginning of the plateau was also reduced. For instance, V- or U-notches with a 0.2 mm depth had a plateau starting around 4.6 mm displacement, while for a 1.5 mm depth, the plateau began at about a 3.0 mm displacement. The difference between the experimental and the FE results during failure may be attributed to how the defect (notch) develops and propagates during the experimental tests and the FE analyses. In the experimental tests, the defect propagated and tore the samples with the increasing imposed loads. Instead, the FE mesh remained continuous throughout the analysis, and no gaps (or voids) were initiated. Hence, the actual rupture of the material, which would demand the discontinuity of the FE mesh, was not modeled. Still, the increasing acting strains progressively reduced the element stiffness to simulate the sample’s failure. This approach modifies the stress field close to the notch tip, particularly given the constraint caused by a sharp curvature radius, possibly altering the failure mechanism development. It is recognized that this is a limitation of the proposed approach that should be investigated in future studies. 

Table 5 compares the loads related to Points (A) to (D) obtained in the experimental tests and those estimated with the FE model considering the V- and U-notches and different depths. Regarding the V-notch, the experimental forces were about 3.4% higher on average, with a standard deviation of 10%, leading to a coefficient of variation of 9.7%. Moreover, the U-notch results indicate experimental values of only 0.1% higher than the FE ones on average, with a standard deviation of 6.9%, resulting in a coefficient of variation equal to 6.9%. Hence, despite some significant specific differences, the FE model results agreed well with the experimental measurements on average.

**Figure 16:**
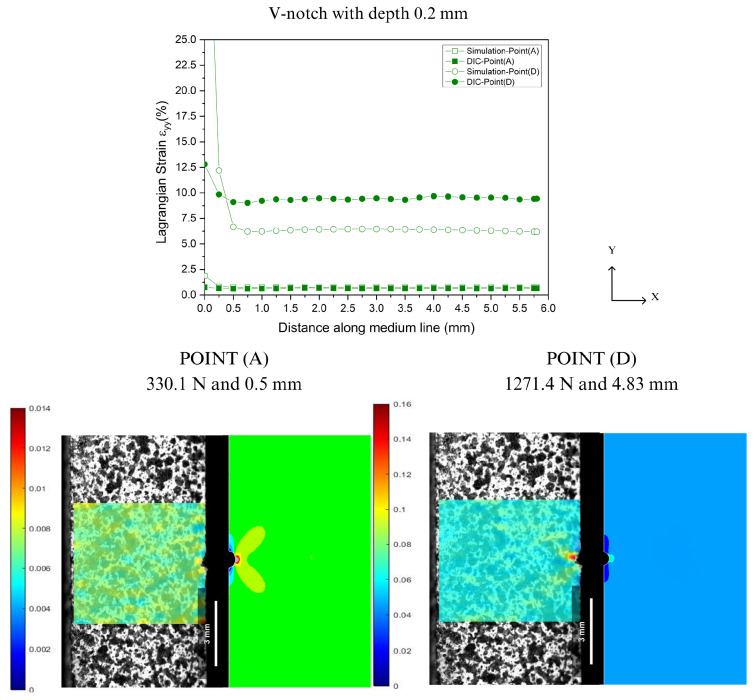
Comparison between DIC technique and FEA at Point A and Point D for V-notch tip with 0.2 mm depth.

**Figure 17:**
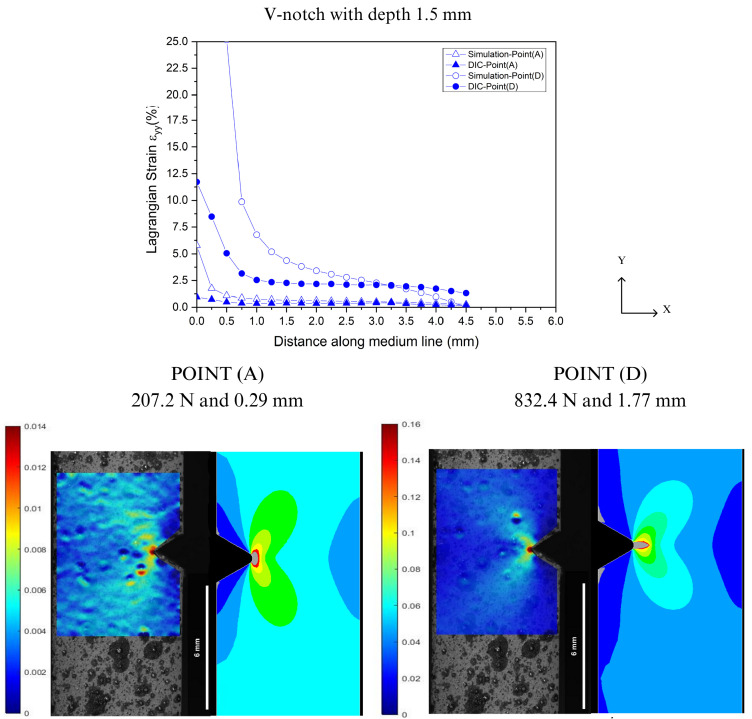
Comparison between DIC technique and FEA at Point A and Point D for V-notch tip with 1.5 mm depth.

**Figure 18:**
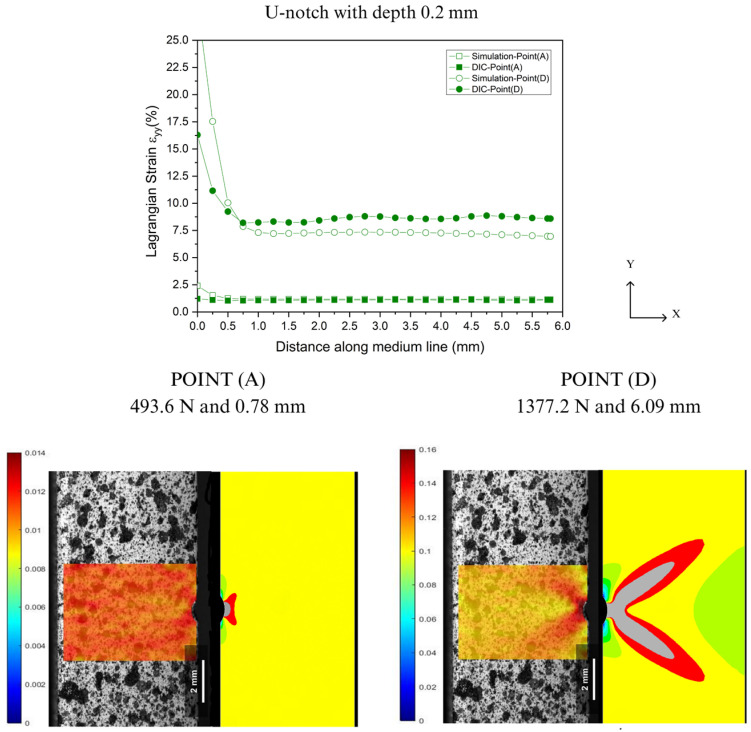
Comparison between DIC technique and FEA at Point A and Point D for U-notch tip with 0.2 mm depth.

**Figure 19:**
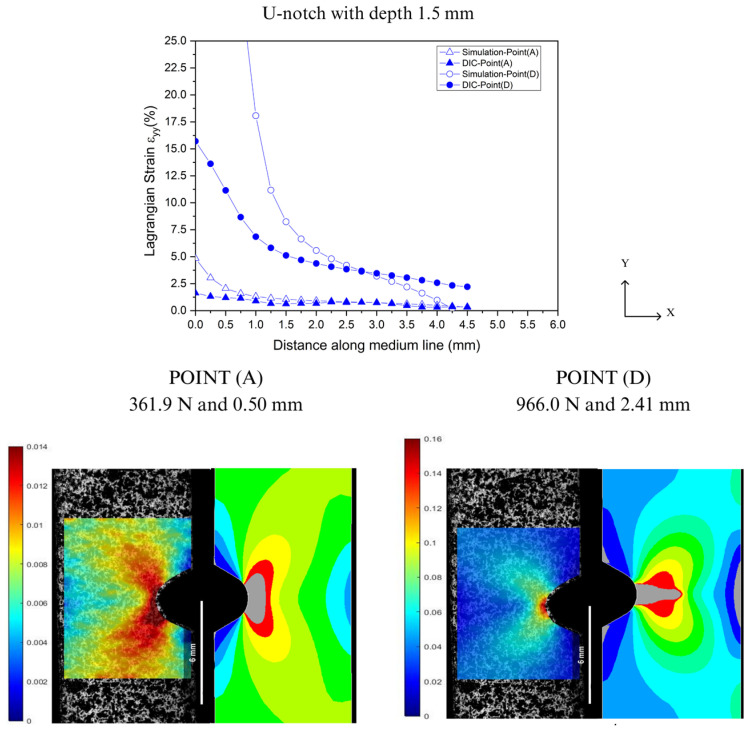
Comparison between DIC technique and FEA at Point A and Point D for U-notch tip with 1.5 mm depth.

In the prediction of localized deformations, Lagrangian strains were extracted along the Y-axis from the notch tip for both DIC and FEA, as illustrated in Figure 4. Figure 16, Figure 17, Figure 18 and Figure 19 show the corresponding results at Point A (the load associated with the elastic region) and Point D (the load related to the yield point criterium), in which the region near the notch tip presents values of higher deformation.

In Figure 16, Figure 17, Figure 18 and Figure 19, the accuracy and precision of the strain maps near the edge of the stress concentrators (below 0.5 mm) are more sensitive to modifying DIC parameters for ductile materials. This observation is consistent with Liu’s report on the assessment of notches [41]. DIC results can be significantly impacted by the quality of speckle patterns, resolution, and discontinuity of the mesh. Moreover, the localized whitening phenomenon at the notch tip, which is usual in PVDF [33], plays an important role in the resolution levels used in FEA and DIC because the numerical model used in the analysis did not consider the whitening effect and viscoelastic behavior typical of PVDF. On the other hand, as the observation point moved away from the notch edge toward Point A (at 0.5 mm), both methods showed good convergence with only a slight difference in results.

## 4. Conclusions

PVDF was machined with U- and V-notches considering four different depths (0.2, 0.6, 1.0, and 1.5 mm). The samples were subjected to tensile tests at 23 °C, and the DIC technique was employed to map the related strains until the samples’ failure. Then, the mechanical (strain gauges) and DIC results were analyzed and compared to the FE models that reproduced the experimental tests, leading to the following conclusions:

- In the experimental tests, the decay of the failure loads was observed with an increase in notch depths and a reduction in the notch radius in both U and V geometries. The decay is associated with an increased three-dimensional stress state at the notch tip.
- The DIC method showed the notch effect on the samples’ deformation by generating strain maps. Severe constraints were detected, and the deformation around the notch tip under tensile loading was quantified.
- The FEA showed similar load vs. displacement curves compared to those obtained experimentally. Hence, the FE model well captured the global behavior of the unnotched and notched samples.
- When comparing the DIC technique and FEA, it was observed that numerical simulation can lead to deviations, possibly due to the continuous approach adopted in the FE models, which may affect the stress field close to the notches. This aspect may be studied in future work. Additionally, the difference in resolution levels between the DIC technique and FEA can influence the results measured for strain around the notch. However, a reasonable convergence between numerical and experimental strain results was observed at points distant 0.5 mm or higher from the defect.

## Figures and Tables

**Table 1:** Notch groups and correspondent depth.

Specimens’ Group	Depth (mm)
Unnotched	---
V-notch	0.2
	0.6
	1.0
	1.5
U-notch	0.2
	0.6
	1.0
	1.5

**Table 2:** Properties of commercial grades of PVDF.

Properties	Values (Unity)
Density	1.78 × 10^5^ kg/m^3^
Poisson’s ratio	0.43
Elastic modulus	1280.15 MPa

**Table 3:** The total number of elements and nodes corresponding to each notch geometry with different depths.

Specimens’ Group	Depth (mm)	Number of Elements	Number of Nodes
V-notch	0.2	8622	29,161
	0.6	9087	27,550
	1.0	9047	27,510
	1.5	9003	27,288
U-notch	0.2	9450	28,794
	0.6	9017	23,760
	1.0	8847	26,860
	1.5	8414	25,547

**Table 4:** Maximum load and displacement to each notch geometry with different depths.

Specimens’ Group	Depth (mm)	Maximum Load (MPa)	Maximum Displacement (mm)
Unnotched		1393.0 ± 31.4	8.0 ± 0.2
V-notch	0.2	1391.4 ± 5.7	8.4 ± 0.2
	0.6	1305.4 ± 30.6	6.1 ± 0.4
	1.0	1197.3 ± 6.9	4.5 ± 0.2
	1.5	1042.1 ± 17.2	3.3 ± 0.1
U-notch	0.2	1447.0 ± 19.5	8.3 ± 0.3
	0.6	1354.0 ± 19.4	7.2 ± 0.3
	1.0	1263.4 ± 23.4	5.6 ± 0.1
	1.5	1184.1 ± 21.5	4.7 ± 0.2

**Table 5:** Comparison of load of notched specimens between FEA-simulated and experimental (Exp.) data.

Specimens’ Group	Depth (mm)	Load (N)							
		Point (A)		Point (B)		Point (C)		Point (D)	
		Exp.	FEA	Exp.	FEA	Exp.	FEA	Exp.	FEA
V-notch	0.2	330.1	279.2	1027.8	1124.5	1240.3	1305.4	1271.4	1322.1
	0.6	380.9	395.8	760.6	881.3	986.4	1104.2	1187.2	1222.1
	1.0	231.7	221.7	500.2	551.5	708.7	800.5	1042.6	1087.8
	1.5	207.2	169.8	609.4	556.2	760.1	732.4	832.4	818.5
U-notch	0.2	493.6	540.9	923.0	1064.6	1218.3	1276.5	1377.2	1325.5
	0.6	427.7	433.3	817.3	937.2	1069.4	1170.2	1248.3	1225.4
	1.0	399.6	396.2	799.9	794.5	1023.7	1022.9	1173.8	1123.6
	1.5	361.9	340.1	667.9	675.0	888.3	891.0	966.0	940.7

## Data Availability

Data are contained within the article.
